# Fifty shades of iron: Unorthodox mechanisms of iron acquisition and utilization in blood-stage *Plasmodium* parasites

**DOI:** 10.1371/journal.ppat.1014030

**Published:** 2026-03-10

**Authors:** Kade M. Loveridge, Paul A. Sigala

**Affiliations:** Department of Biochemistry, University of Utah, Salt Lake City, Utah, United States of America; Joan and Sanford I Weill Medical College of Cornell University, UNITED STATES OF AMERICA

## Abstract

*Plasmodium falciparum* parasites cause severe human malaria and depend on iron for essential metabolic processes during all phases of their complicated lifecycle, including when growing in human red blood cells (RBCs). Despite decades of study, the major pathways by which malaria parasites access, distribute, and regulate iron during blood-stage infection remain incompletely defined. The parasite genome lacks many canonical transporters, storage proteins, reductases, and regulatory circuits that are essential for maintaining iron homeostasis in model organisms. Emerging evidence suggests that blood-stage parasites employ unconventional strategies to maintain iron homeostasis. In this review, we synthesize current knowledge of how blood-stage *P. falciparum* manages iron, from initial uptake through cellular distribution to utilization, highlighting the key proteins and pathways that shape parasite iron metabolism. We also identify major unanswered questions that will guide future efforts to understand and therapeutically target this essential aspect of *Plasmodium* biology.

## 1. Introduction

Iron is essential for nearly all forms of cellular life. Its abundance in Earth’s crust and its ability to oxidatively cycle between ferrous (Fe^2+^) and ferric (Fe^3+^) states have made it the most widely used transition metal in biological systems [1]. Iron underpins diverse cofactors that modulate protein reactivity and redox chemistry, including heme, iron–sulfur (Fe–S) clusters, diiron centers, and mononuclear iron [2]. Yet the same properties that make iron indispensable also render it potentially cytotoxic, since excess labile iron can drive Fenton chemistry that results in reactive oxygen species [3]. To balance this duality, organisms have evolved tightly coordinated systems for iron uptake, trafficking, storage, and detoxification [4,5].

A critical axis that exemplifies this exquisite balance is at the host–pathogen interface, where iron availability can shape infection outcomes and drive evolutionary arms races [6]. *Plasmodium falciparum*, the most virulent cause of human malaria, depends on iron throughout its complex lifecycle, which spans two hosts and multiple developmental stages. Following inoculation into humans by an infected *Anopheles* mosquito, parasites migrate to the liver, where they proliferate silently for one to two weeks before releasing merozoites into the bloodstream. These merozoites invade iron-rich red blood cells (RBCs), replicating in 48-hour cycles that culminate in hemolysis and the release of progeny parasites that perpetuate blood-stage infection. The cycle is completed when another mosquito ingests infected RBCs during a blood meal, enabling transmission to a new host [7].

Understanding how parasites access iron in the blood stage is of particular interest, because this stage is responsible for all clinical disease and, in severe cases, can result in end-organ failure and death [8]. When growing and replicating in RBCs, the parasite depends on iron for conserved processes such as DNA synthesis and mitochondrial electron transport chain (ETC), as well as for specialized functions, such as isoprenoid biosynthesis and maintenance of its unique plastid organelle, the apicoplast [9,10]. Host iron status also has clinical relevance for malaria, since host anemia protects against severe malaria, while iron supplementation increases parasite burden. This interdependence provides evidence that iron availability directly or indirectly shapes parasite load and disease progression [11,12]. Despite recognition of this central relationship between iron and malaria, major gaps remain in our understanding of how *P. falciparum* accesses, senses, regulates, and utilizes iron within the RBC.

In model systems such as yeast and mammals, decades of work have revealed complex networks that regulate iron uptake, distribution, and storage [5,13,14]. These organisms rely on transporters, chaperones, storage proteins, and transcriptional regulators that maintain iron homeostasis across fluctuating conditions. However, many proteins that canonically function to buffer cellular iron concentrations have no clear homologs in parasites of the phylum Apicomplexa, including *P. falciparum*. This absence, coupled with the extensive evolutionary divergence between *Plasmodium* and most model organisms, suggests that parasites rely on unconventional or entirely novel mechanisms to regulate iron.

It is one of the enduring “ironies” of malaria biology that major mechanisms of iron homeostasis in blood-stage parasites remain sparsely defined, despite these pathogens residing in the most iron-rich cell in the human body. This gap raises broader questions about how the parasite meets its iron requirements, balances intracellular stores, and adapts to its unique intracellular niche. In this review, we synthesize current knowledge of iron biology in blood-stage *P. falciparum*—focusing on iron acquisition, transport, regulation, and utilization. By synthesizing current findings and identifying key open questions, we hope to stimulate future research into the many unexplored facets of malaria parasite iron regulation and metabolism.

## 2. Iron acquisition

### 2.1. Malaria parasites rely primarily on intracellular RBC iron

A central question is whether blood-stage parasites depend on extracellular iron (beyond the RBC) or if RBC-derived iron is sufficient to meet their nutritional needs. Early models favored parasite uptake of serum iron (e.g., transferrin), partly because host anemia correlates with reduced parasitemia [15,16]. However, iron chelators that cannot cross the infected RBC (iRBC) membrane do not affect parasite growth, whereas deferoxamine (DFO), which effectively crosses the iRBC plasma membrane (but not uninfected RBCs) kills parasites rapidly at ~10 µM [17,18]. Likewise, parasites cultured in iron-depleted medium grow normally, and direct assays confirm poor transferrin uptake [19,20]. These findings argue against serum iron as a primary or necessary source.

Although some studies suggest transferrin iron can be accessed under specific conditions, its physiological importance and potential mechanism remain unclear, considering that parasites do not require serum transferrin when cultured ex vivo [12]. More recently, X-ray fluorescence microscopy revealed that iRBCs do not increase the total amount of cellular iron compared to uninfected RBCs (unlike other trace metals), further supporting a model in which parasites rely on pre-existing RBC iron rather than internalizing iron from extracellular pools [21].

Within RBCs, two major reservoirs of iron could support parasite metabolism. The dominant iron source is hemoglobin, an iron metalloprotein that is internalized in bulk by parasites during intraerythrocytic infection and delivered to the lysosome-like food vacuole (FV) for proteolysis, which liberates copious amounts of heme [22]. Other host metalloproteins, such as catalase, are also likely trafficked to the FV and digested similarly [23]. The second RBC reservoir is the cytosolic labile iron pool (LIP), consisting of submicromolar Fe^2+^ loosely bound to ligands such as glutathione, citrate, and ATP [24]. Importantly, because the LIP is not stably coordinated, it can be bound and sequestered by iron chelators such as DFO. This LIP differs from hemoglobin, where iron is protected from sequestration by tight coordination within heme surrounded by the protein scaffold.

To test if parasites depend on the RBC LIP, several studies osmotically loaded RBCs with concentrations of DFO derivatives that far exceeded the estimated LIP and normal DFO EC_50_ value [18,25]. Surprisingly, only RBCs loaded with extremely high concentrations of DFO resulted in impaired parasite growth. The major implication of these experiments is that parasites can survive without accessing the RBC LIP and that iron chelators like DFO require internalization into parasites to exert their cytotoxic effects. Therefore, the dominant parasite iron pool does not appear to arise directly from the RBC LIP but rather from non-labile RBC iron sources that are metabolized into a parasite-accessible LIP.

However, there is evidence that parasites can access the RBC LIP, even if this pool is not strictly essential. In time-course rescue experiments, growth of synchronized parasites treated with DFO was rescued by adding exogenous iron to the medium up to 36 h after treatment, implying most simply that the supplemented iron traversed the RBC, parasitophorous vacuole, and parasite plasma membranes to restore intracellular iron availability [26]. This observation suggests that parasites can utilize labile iron (at least when supplied in excess). Consistent with this mechanism, loss of RBC ferroportin (the iron exporter on the RBC membrane) increases the RBC LIP and has been associated with higher mortality in *Plasmodium*-infected mice and is suggested to reflect enhanced parasite growth [27]. Thus, while non-labile iron appears primary, the RBC LIP may represent an auxiliary source that can be exploited by parasites under certain conditions.

### 2.2. Labile iron generation within the FV

**Fig 1:**
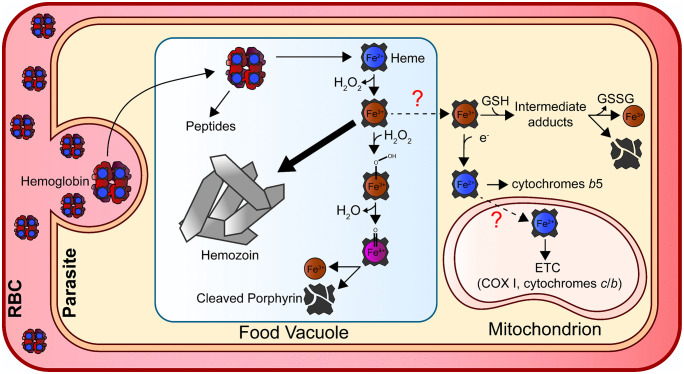
Schematic representation of proposed mechanisms for non-enzymatic heme degradation leading to the release of free iron. The peroxide-mediated process in the FV is shown in greater detail, as in vitro studies have more thoroughly characterized its reaction intermediates compared with glutathione-mediated mechanisms, which remain less defined. The inferred redox state of heme (oxidized or reduced) reflects the overall redox environment of the subcellular compartment indicated in the diagram. Heme within the FV, or heme that is subsequently scavenged for use in other organelles, is assumed to originate primarily from hemoglobin digestion. However, alternative routes of heme acquisition are possible (and may include uptake and digestion of other red cell iron proteins).

Blood-stage parasites take up large amounts of hemoglobin from the RBC cytosol and traffic it to the FV, where prodigious amounts of free heme are liberated [22,28]. Most heme is biomineralized into insoluble hemozoin crystals [22], which rapidly tumble in the FV. This motion is stimulated by catalytic breakdown of hydrogen peroxide by surface-exposed metals on hemozoin crystals that may reflect labile iron released by nonenzymatic heme breakdown [29]. Indeed, not all RBC heme is sequestered into hemozoin. Because the parasite’s heme biosynthesis pathway is dispensable in blood-stage parasites, these organisms must employ an as-yet unidentified alternative mechanism to obtain heme for essential proteins like mitochondrial cytochromes (Fig 1) [30,31]. While scavenged heme is expected to be derived from the FV, there are no homologs of known heme transporters (e.g., HRG1, FLVCR1) that have been identified based on sequence identity [32]. However, the high concentration of heme in the FV may allow for passive low-level leakage, potentially eliminating the need for a dedicated transporter.

Many organisms acquire heme and enzymatically degrade it using a highly conserved family of proteins known as heme oxygenases, which release iron for metabolic utilization [33]. Although *Plasmodium* encodes a heme oxygenase homolog (Pf3D7_1011900), this protein lacks canonical heme-degrading activity and instead plays a key adaptive role in transcription of the apicoplast genome [34]. In the absence of a conserved enzymatic pathway for heme degradation, there have been multiple proposed mechanisms for iron generation from scavenged heme.

The FV has been proposed as the most likely site of labile iron generation by blood-stage *Plasmodium* due to several key factors that include its high iron content, its functional similarity to lysosome-like organelles that mediate iron homeostasis in other eukaryotes, and recent evidence suggesting this compartment is the entry portal for essential parasite iron (discussed in Section 3) [22,26]. The current model posits that heme is degraded in the FV via a non-enzymatic, peroxide-assisted mechanism that is well described in scientific literature (Fig 1) [35]. Although this mechanism is less efficient than enzyme-catalyzed heme degradation, the high concentration of heme within the FV and modest parasite need for nutritional iron may compensate for this inefficiency. Moreover, in vitro studies have demonstrated that heme can undergo non-enzymatic degradation under in vitro conditions that mimic the FV environment [36]. However, direct in vivo evidence for this process is still lacking, and it remains unclear whether this process is the primary mechanism by which labile iron is generated in the FV.

### 2.3. Other proposed mechanisms for intracellular iron production

Reduced glutathione (GSH) has been shown to degrade heme and release free iron in vitro at pH values ≥7.0, and has been proposed as an iron-generating mechanism in the parasite cytosol (Fig 1) [37,38]. However, no in vivo evidence supports cytosolic heme degradation as a major contributor to parasite iron pools. While FV-derived iron appears essential for fitness, cytosolic production remains uncertain [26].

## 3. Iron transport

**Fig 2:**
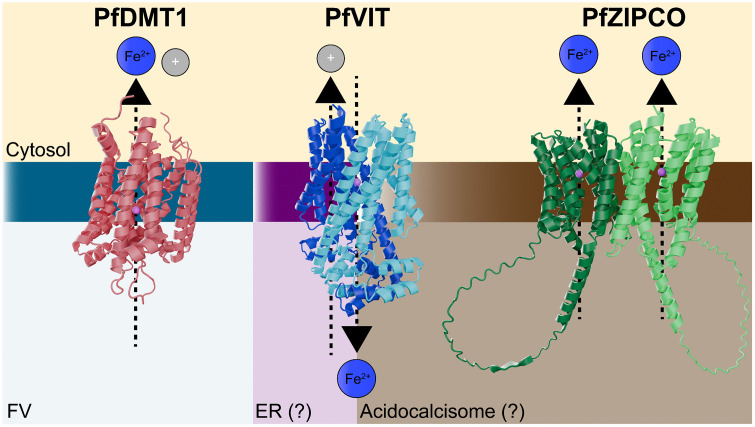
Predicted AlphaFold structures of PfDMT1, PfVIT, and PfZIPCO. Each model was generated in the presence of a Mn^2+^ ion, as AlphaFold currently does not support Fe^2+^ modeling and the two ions have comparable atomic radii. By homology with related transporters in other organisms, PfVIT and PfZIPCO are predicted to form dimers. PfVIT dimers are thought to generate a single shared transport channel, whereas PfZIPCO dimers are expected to contain two independent channels, one within each monomer. The directionality and associated ions of transport are indicated by the arrows and labeled ions, respectively.

Regardless of the iron source, all organisms must possess efficient mechanisms to move charged iron ions across membranes, as they cannot freely diffuse through lipid bilayers [13,14]. In *P. falciparum*, only three transporters have experimental evidence supporting their substantial roles in iron transport, and only PfDMT1 appears to mediate iron acquisition directly from the host RBC (Fig 2). This section will discuss what is known about each validated iron transporter, propose potential unstudied iron transporters, and highlight areas where iron transport is poorly understood in blood-stage malaria parasites.

### 3.1. DMT1/NRAMP2

Divalent metal transporter 1 (DMT1, which is also known as Natural Resistance-Associated Macrophage Protein 2, NRAMP2), is the main gateway for cytosolic iron uptake in most eukaryotes, mediating both dietary absorption and iron release from endosomes and lysosomes [39]. As a proton-coupled, voltage-dependent carrier, it functions optimally in acidic compartments but requires ferric reductases (Dcytb, STEAP3, FRE6) to generate the Fe^2+^ substrate it transports [13,14,40]. In addition to moving iron into the cytosol, DMT1 also helps load iron into mitochondria through transient “kiss-and-run” contacts with endo/lysosomes. This poorly understood process has broad relevance, as its disruption is linked to human pathologies, like cancer [41].

The *P. falciparum* homolog, PfDMT1 (PF3D7_0523800, Fig 2), was identified more than 20 years ago, but its function was only recently investigated. PfDMT1 localizes to the FV membrane, and knockdown (KD) experiments show it is essential for blood-stage growth [26,42]. Loss of PfDMT1 causes severe, irreversible, and pleotropic cellular defects within 24 hours. Importantly, excess iron does not rescue parasites from complete PfDMT1 KD, in contrast to the iron chelator DFO [26]. By comparison, partial KD (~50%) is highly detrimental to parasite growth but can be selectively rescued by iron supplementation (but not other metals). A similar rescue by exogenous iron was observed when *P. yoelii* DMT1 expression was reduced by half [26,43]. Together, these findings underscore two points: (1) iron is the physiologically dominant substrate of *Plasmodium* DMT1 proteins, and (2) the FV is a major site of iron uptake in blood-stage parasites, where PfDMT1 serves as a non-redundant gatekeeper.

Earlier proposals suggested that the FV membrane chloroquine resistance transporter (CRT) mediated iron transport, but this hypothesis was not supported by subsequent studies [44,45]. Moreover, the lethality of PfDMT1 knockdown indicates that CRT cannot substitute for this essential transporter, further arguing against a significant role in iron uptake [26]. PfDMT1 KD also led to defects in the mitochondrial ETC and in apicoplast biogenesis, both iron-dependent processes [26]. These phenotypes underscore the central role of PfDMT1, though they remain indirect measures of iron metabolism. Without direct methods to assess compartment-specific iron, it remains unclear whether PfDMT1 supplies all cellular iron pools or whether some compartments can be maintained through partially redundant or alternative iron-acquisition pathways.

The apicoplast biogenesis defect observed upon PfDMT1 knockdown persists even when the isoprenoid precursor synthesis pathway is chemically complemented, indicating that iron is required for apicoplast functions beyond SUF-mediated Fe–S biogenesis. Two non-heme, non-Fe–S enzymes predicted to utilize iron, peptide deformylase (PF3D7_0907900) and methionine aminopeptidase (PF3D7_0804400), are detected in the apicoplast proteome. These enzymes likely catalyze N-terminal processing of nascent proteins [46]. Their predicted essentiality, combined with the failure of isoprenoid precursors to rescue and restore apicoplast integrity, supports a model in which iron delivery to the apicoplast sustains core protein maturation chemistry that cannot be bypassed by restoring isoprenoid outputs [26,47,48]. It is also possible that PfDMT1 KD disrupts iron-dependent enzymes outside the apicoplast that are required for organelle biogenesis.

A key unresolved question is how *Plasmodium* maintains iron in the reduced Fe^2+^ state for transport by PfDMT1 and other transporters, as apicomplexans lack recognizable ferric reductases (e.g., FRE6, STEAP3, Dcytb) [49]. In blood stages, host metabolites such as glutathione or ascorbate might be internalized during hemoglobin uptake and act as reductants [50,51]. The uptake of massive amounts of Fe^2+^ hemoglobin could also feasibly provide reducing equivalents for liberated Fe^3+^ [52]. However, these models cannot explain how iron is reduced in related apicomplexans that conserve DMT1 but do not live inside RBCs. Defining how *Plasmodium* and related apicomplexans regulate the iron redox state, therefore, remains a central challenge for understanding apicomplexan biology.

PfDMT1 is unusually divergent from DMT1 homologs in both other eukaryotes and fellow apicomplexans [26]. Remarkably, *Toxoplasma* DMT1 shares greater similarity with human DMT1 (43%) than with PfDMT1 (34%). *Plasmodium* sequences show substantial alterations in conserved metal-binding motifs, and these changes are predicted to influence transport properties in ways that remain poorly understood. These differences suggest the possibility that *Plasmodium* has rewired a canonical iron transporter to meet its specialized metabolic demands in the unique environment of the RBC.

### 3.2. VIT

Vacuolar iron transporters (VITs), known as CCC1 in yeast, are conserved in plants, fungi, archaea, and protozoa but absent in animals [53]. They import Fe^2+^ into acidic organelles such as vacuoles or acidocalcisomes, typically as proton–metal antiporters. In yeast and plants, this activity protects against cytosolic iron overload by sequestering excess iron [53].

In *Plasmodium*, VIT homologs have been studied in both *P. berghei* and *P. falciparum* (PF3D7_1223700, Fig 2), but their localization remains debated. PbVIT unexpectedly localized to the ER rather than to the FV, with the ER environment unlikely to support iron transport via proton-driven antiport [54]. Nevertheless, PbVIT expression rescued yeast CCC1 mutants, and isolated PbVIT-expressing yeast vacuoles imported iron, providing functional evidence of its capacity to act as an iron importer [54]. In contrast, PfVIT was found mainly in discrete cytoplasmic vesicles, proposed to be acidocalcisomes [55]. This alternative localization would align with the evolutionary history of VIT and its typical energetic transport requirements. However, parasite markers of acidocalcisomes are not readily available to test this hypothesis [55]. Whether the observed differences in localization between *P. berghei* and *P. falciparum* are artifactual or represent a species-specific difference is a key barrier to understanding *Plasmodium* VIT function. Adding to this complexity, a recent study of the *T. gondii* VIT found some evidence that TgVIT localizes to the plant-like vacuole but also includes a population localized to acidocalcisomes [56].

Although VIT is predicted to be dispensable in blood-stage *P. falciparum*, functional studies reinforce its physiological importance for growth inside RBCs [47]. Knockout (KO) of PbVIT results in impaired parasite growth in both liver and blood stages and enhanced sensitivity to iron overload [54]. PbVIT KO parasites accumulate higher levels of labile iron and display reduced capacity to cope with excess iron stress, underscoring the role of VIT in modulating cellular iron levels [54].

### 3.3. ZIPCO

ZIP (Zrt- and Irt-like Proteins) are a broad family of metal transporters conserved across eukaryotes and bacteria [57]. While these proteins are primarily recognized as zinc transporters, a distinct evolutionary subset has adapted to also transport iron [58]. *Plasmodium* encodes two ZIP homologs: ZIP1 (PF3D7_0609100) and ZIPCO (ZIP domain–containing protein, PF3D7_1022300, Fig 2). Phylogenetic and functional analyses support ZIP1 functioning exclusively as a zinc transporter [59]. In contrast, ZIPCO shares comparatively less sequence identity with canonical ZIP proteins. Recently, the *T. gondii* homolog (termed ZFT) has been shown to play a central role in iron acquisition [60,61].

PbZIPCO is dispensable in both blood and mosquito stages, and genome-wide knockout studies likewise predict that PfZIPCO is not required during the blood stage [47,62]. However, prior studies report that PbZIPCO is critical for iron acquisition during liver-stage development [62]. It is also noteworthy that PfZIPCO expression is massively upregulated during mosquito growth, suggesting increased importance in this lifecycle stage.

Localization studies in liver stages detected signal at the parasite plasma membrane along with internal puncta [62]. In contrast, blood-stage *P. falciparum* studies suggest PfZIPCO localizes primarily to internal foci resembling those observed for PfVIT that may be acidocalcisomes [55]. Notably, among the three *Plasmodium* iron transporters studied to date (DMT1, VIT, and ZIPCO), ZIPCO shows the strongest transcriptional response to changes in iron availability [55].

### 3.4. Interplay of DMT1, VIT, and ZIPCO in iron handling

The available data for PfDMT1, VIT, and ZIPCO suggest that *Plasmodium* has rewired conserved transporters into a distinctive iron-handling network. In plants and yeast, VIT imports iron into vacuoles while DMT1 later remobilizes it. However, no identifiable FV iron importer has been found in *Plasmodium*, and the parasite lacks ferritin or any analogous high-capacity cytosolic iron-storage protein [49,53]. Instead, if the FV generates iron primarily through non-enzymatic heme breakdown (a process that may not be easily regulated), PfDMT1 may function more like a “leaky faucet” than a tightly controlled valve. Lacking both a mechanism to return iron to the FV and a ferritin-like storage system, the parasite would need to sequester excess labile iron elsewhere. The acidocalcisome is a compelling candidate for buffering excess iron in parasites. Proteomic experiments in trypanosomes have identified VIT as localized to acidocalcisomes, consistent with a role in iron sequestration and lending plausibility to the proposed localization of PfVIT in *Plasmodium* [63].

**Fig 3:**
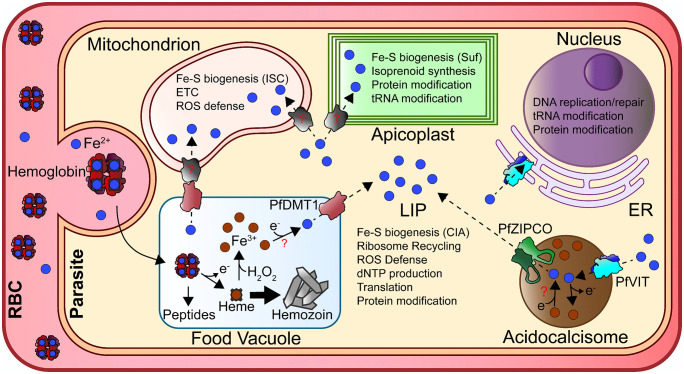
Schematic representation of proposed mechanisms for iron acquisition and handling in blood-stage *Plasmodium falciparum* parasites, incorporating pathways with at least some experimental support.

A critical unresolved question is whether iron stored in acidocalcisomes can be remobilized for metabolism. Studies in trypanosomes and other protists have demonstrated that the iron content of these organelles fluctuate in response to changes in cellular iron status [64,65]. However, no dedicated mechanism for iron export from acidocalcisomes has been identified in any system, whether by direct transport across the organelle membrane or by fusion with a lysosome-like compartment to release stored ions, making this a central open question in acidocalcisome biology [64]. In the context of *Plasmodium*, one potential hypothesis is that PfZIPCO mediates such efflux, potentially partnering with PfVIT to enable bidirectional flux (Fig 3) [55]. Clarifying whether PfVIT and PfZIPCO colocalize will be key to testing this hypothesis and understanding how parasites manage an appropriate LIP concentration.

### 3.5. Putative transporters

Recent work has provided evidence of the importance of DMT1, VIT, and ZIPCO, but these proteins alone are unlikely to account for the full delivery of iron to all subcellular compartments where it is needed. This gap is most evident for the mitochondrion and apicoplast, both of which are expected to have heightened iron requirements that include Fe–S biogenesis pathways in both organelles [14,66].

The mitochondrion is one of the most iron-demanding organelles in the cell, and iron must cross both the outer and inner mitochondrial membranes (OMM/IMM) to support the ETC and Fe–S cluster biogenesis. The OMM is highly permeable due to voltage-dependent anion channels, which allow diffusion of a range of small iron–ligand complexes. In contrast, the IMM is far more selective and requires dedicated, high-affinity iron importers [14]. One of the most compelling candidates in *Plasmodium* is the homolog of mitoferrin (MFRN; PF3D7_0905200), the conserved IMM iron transporter in yeast and mammals [67]. The *Plasmodium* MFRN homolog is predicted to be essential and has been localized to the mitochondrion, making it a strong candidate for mediating mitochondrial iron uptake in the parasite [47,55].

How the apicoplast acquires iron remains unresolved. Because the apicoplast is a product of secondary endosymbiosis, it has four membranes that create a major barrier for metal import [9]. One candidate is ATP10 (PF3D7_0727800; Table 1), annotated as a putative Mn^2+^ transporter that may support the function of the putative Mn^2+^ metalloprotein, DXR (1-deoxy-D-xylulose 5-phosphate reductoisomerase), the first committed enzyme of the isoprenoid biosynthesis pathway [68,69]. Because Fe^2+^ and Mn^2+^ have similar atomic radii and ligand preferences, transporters for one metal often show promiscuity for the other [70]. Therefore, if ATP10 is also capable of transporting Fe^2+^, its predicted essentiality could reflect a dual role in supplying both Mn^2+^ and Fe^2+^ to apicoplast metalloproteins required for isoprenoid synthesis (e.g., DXR, IspG, IspH) [9,71]. Still, no direct evidence supports this model, leaving the physiological role of ATP10, the potential function of additional transporters to mobilize iron across four membranes, and the overall mechanism of apicoplast iron acquisition unknown.

**Table 1:** Putative and characterized Fe–S cluster carrier proteins in *Plasmodium falciparum*. Proteins that have been directly localized, or for which localization has been demonstrated in other apicomplexans, are annotated with an “l.” Proteins are classified as dispensable, fitness-conferring, or essential based on insertional mutagenesis studies [47,48,92], or, when available, on direct experimental evidence from inducible knockdown or knockout systems in *P. falciparum*, which are annotated with a “d.”.

Fe–S Carriers					
Protein	Gene ID	Localization	Essentiality		
			*P. falciparum*	*P. knowlesi*	*P. berghei*
GLP1	PF3D7_0304500	l-Cytosol	Fitness cost	Essential	NA
GLP2	PF3D7_0606900	l-Cytosol	Essential	Dispensable	Dispensable
GLP3	PF3D7_0709200	l-Mito	Fitness Cost	Fitness Cost	Dispensable
BolA	PF3D7_0515800	Cytosol	Essential	Fitness Cost	NA
CDGSH1	PF3D7_0302700	l-Cytosol/Mitochondrion (?)	Essential	Fitness Cost	NA
CDGSH2	PF3D7_0416700	Mitochondrion	Dispensable	Dispensable	Dispensable
ApiCOX13	PF3D7_1022900	l-Mitochondrion	Dispensable	NA	Essential
IscA1	PF3D7_0207200	l-Mitochondrion	Essential	Essential	Essential
IscA2	PF3D7_0322500	l-Mitochondrion	Dispensable	Fitness Cost	NA
NAR1	PF3D7_0614200	Cytosol	Essential	Essential	NA
HC101	PF3D7_1128500	l-Cytosol	Essential	Essential	Essential
CIA1	PF3D7_1209400	Cytosol	Fitness Cost	Essential	Essential
CIA2	PF3D7_0703100	Cytosol	Essential	Essential	NA
SufA	PF3D7_0522700	l-Apicoplast	d-Dispensable	Dispensable	Dispensable
NfuApi	PF3D7_0921400	l-Apicoplast	d-Dispensable	Dispensable	Dispensable

The physiological substrates of most *Plasmodium* transporters remain uncharacterized, including those of proteins implicated in drug resistance, such as multidrug resistance protein (MDR) 1 and 2 [72]. Mutations in MDR2 have been linked to heavy metal toxicity resistance, potentially implicating a role in metal transport [73]. Given the large number of uncharacterized transporters, additional proteins critical for mediating iron translocation across discrete membranes in *Plasmodium* likely await discovery.

## 4. Iron regulation and trafficking

### 4.1. Iron regulation

In yeast and mammals, iron availability is tightly regulated through global transcriptional programs [13]. In yeast, this coordination involves the iron regulon that is activated under low iron by Aft1/2 and the iron-sparing response that is induced under high iron by Yap5 [74]. Together, these pathways coordinate the expression of iron uptake, storage, and utilization proteins. *Plasmodium* lacks homologs of these proteins, consistent with general understanding that transcriptional control is not a dominant regulatory mechanism in *Plasmodium* and that most parasite transcription factors characterized so far function in sexual gametocyte development [75].

In mammals, low iron triggers post-transcriptional regulation through iron response elements (IREs) in untranslated mRNA regions [5]. These stem-loops bind iron regulatory proteins (IRPs), stabilizing transcripts when located in the 3′ UTR or repressing translation when in the 5′ UTR [76]. Only a limited set of genes are controlled in this way, and IRE-prediction algorithms often yield false positives [77,78]. Early work suggested that IRE-like motifs were observed in *Plasmodium*, but the IRE/IRP axis has since been shown to be metazoan-specific [78,79]. In *T. gondii*, a predicted IRE-like stem-loop in the ZFT (ZIPCO homolog) gene altered transcript abundance in response to iron but showed no interaction with IRP-like proteins. This observation underscores that this system is mechanistically distinct and suggests that any similarities with the IRE/IRP are likely products of convergent evolution [61]. Although only *T. gondii* ZFT harbors a predicted IRE-like stem-loop, it is interesting that both ZFT and *P. falciparum* ZIPCO transcripts show pronounced responses to iron levels [55,61]. Clarifying whether these parallel effects reflect a shared or distinct regulatory strategy will be key to unraveling transcriptional responses to changing iron availability that may be general to apicomplexan organisms.

By contrast to ZIPCO homologs, *Plasmodium* proteins such as DMT1 and VIT show inconsistent transcriptional responses to iron, suggesting that any regulation strategies may occur at translation or protein turnover rather than transcription or transcript stability [26,55]. Thus, while ZIPCO stands out as an iron-responsive transcript, the broader mechanisms by which blood-stage parasites might sense and adapt to iron remain poorly defined.

### 4.2. Iron trafficking/sensing

In most cells, a critical fraction of iron exists in the dynamic LIP, which is largely Fe^2+^ loosely bound to ligands such as glutathione, citrate, ATP, and small peptides. Of these interactors, glutathione is vital as it binds and buffers transient Fe^2+^ spikes and has been shown to mediate Fe–S cluster delivery in concert with protein complexes [80]. *Plasmodium* maintains high glutathione levels for redox balance and metabolism, making it a likely contributor to iron buffering and trafficking [50].

Identifying soluble iron carriers that bind free Fe^2+^ has been particularly challenging in *Plasmodium*. In animals, the Poly-C binding protein (PCBP) family comprises multifunctional proteins that both bind RNA and act as cytosolic iron chaperones, where they have been shown to mediate iron loading into ferritin and to deliver Fe^2+^ to cytosolic enzymes [4]. In contrast, *Plasmodium* PCBP-like proteins retain the RNA-binding domains but lack the conserved iron-binding residues, supporting the idea that the iron-delivery function of PCBPs arose later as a metazoan-specific adaptation.

A striking feature of the *Plasmodium* genome is the absence of frataxin, a nearly universal mitochondrial protein that donates iron to ISC machinery and is essential for Fe–S biogenesis in most organisms [81]. Other apicomplexans, including *Toxoplasma*, *Neospora*, and even *Cryptosporidium* (which retains only a mitosome dedicated to Fe–S synthesis), have preserved divergent frataxin homologs [82]. Conversely, the loss of frataxin is observed in *Plasmodium*, certain *Theileria* species, and *Babesia microti*.

This absence raises the question of how *Plasmodium* supplies iron for Fe–S biogenesis. Many apicomplexans that lack a frataxin homolog instead encode a homolog of bacterial IscX (PF3D7_1361600), which in prokaryotes can bind iron and has been proposed to donate it for Fe–S assembly [83]. The conserved retention of IscX in *Plasmodium* and a small subset of related apicomplexans initially made it a compelling candidate to substitute for frataxin. However, *Plasmodium* IscX homologs lack the acidic residues required for iron coordination, and the recombinant protein shows no evidence of iron binding, making this role unlikely [84].

A more plausible alternative is IscA1, a small mitochondrial protein conserved across eukaryotes. IscA proteins bind mononuclear iron with high affinity, transfer it to IscU for cluster assembly, and can transiently coordinate 2Fe–2S clusters for delivery to client proteins [85]. *P. falciparum* encodes two homologs, IscA1 and IscA2 (PF3D7_0207200, PF3D7_0322500, Table 1), but only IscA1 retains the conserved cysteines required for mononuclear iron binding [84]. Consistent with this retention, recombinant PfIscA1 binds Fe^2+^ in vitro, whereas PfIscA2 does not [84]. Genetic evidence also points to essentiality for IscA1, although whether its critical function lies in iron donation, Fe–S transfer, or another role remains untested.

Unlike mononuclear iron chaperones, *Plasmodium* encodes multiple homologs of Fe–S cluster carriers that transiently bind and transfer these cofactors to client proteins, many of which remain poorly studied in this parasite (Table 1). Several of these candidates function in other organisms not only to deliver clusters but also as key regulators of iron balance and redox status. Glutaredoxins (Grxs) and NEET proteins are particularly noteworthy examples, given their established roles in coupling Fe–S biogenesis with iron sensing, redox control, and organelle communication [80,86].

In yeast and mammals, cytosolic Grxs bind 2Fe–2S clusters with glutathione and form complexes with BolA proteins, which both distribute clusters to cytosolic and nuclear enzymes and signal iron sufficiency to regulatory pathways [2]. This dual role distinguishes Grxs as not merely chaperones but also sensors that tune iron metabolism to the cellular redox and glutathione status [80]. In mitochondria, Grxs such as yeast Grx5 act as scaffolds that cooperate with the ISC pathway to assemble and deliver clusters to downstream clients. Loss of Grx5 leads to severe defects in Fe–S protein maturation, mitochondrial iron overload, and oxidative stress, underscoring the central role of this family in balancing iron utilization and signaling [87].

*P. falciparum* encodes three glutaredoxin-like proteins (GLPs) (Table 1). PfGLP1 (PF3D7_0304500) and PfGLP2 (PF3D7_0606900) are predicted cytosolic proteins, while PfGLP3 (PF3D7_0709200) is a mitochondrial protein [88]. All three proteins contain CGFS-type or related active sites predicted to bind 2Fe–2S clusters with glutathione, though direct biochemical experiments in the parasite have not been conducted. By analogy to other systems, the cytosolic GLPs may form 2Fe–2S GLP–BolA complexes with the BolA homolog (PF3D7_0515800), while the mitochondrial GLP may serve as a Grx5-like scaffold in cooperation with the ISC machinery, as shown in *Neospora caninum* [81,87,89]. These features make the GLPs putative candidates for linking Fe–S biogenesis to iron sensing in *Plasmodium*.

A second putative class of Fe–S chaperones in *Plasmodium* are NEET proteins, which coordinate redox-labile 2Fe–2S clusters through the signature CDGSH domain using three cysteines and one histidine [90]. This unusual ligation confers both cluster-transfer capability and strong sensitivity to redox and pH changes. In other systems, NEET proteins are positioned at critical contact sites with the outer mitochondrial membrane, ER–mitochondria junctions, and chloroplasts, where they act as buffers of labile iron, donors of Fe–S clusters, and regulators of inter-organelle communication [86]. In these systems, disruption of NEET function has broad consequences for iron metabolism. Knockdowns or mutations destabilize [2Fe–2S] cluster handling, leading to mitochondrial and cytosolic iron overload, ROS accumulation, and oxidative damage [90]. These defects extend beyond simple redox imbalance, influencing organelle morphology, trafficking, and metabolic integration [86].

*P. falciparum* encodes at least three NEET-like proteins. Two candidates, CDGSH1 (PF3D7_0302700) and CDGSH2 (PF3D7_0416700), resemble classical single-domain NEET proteins [90]. A third candidate, PfApiCox13 (PF3D7_1022900), is more unusual in that it combines two CDGSH domains with a Cox13-like module predicted to contribute to the structural stabilization of complex IV in the mitochondrial ETC. The presence of two CDGSH domains in PfApiCox13 is reminiscent of mammalian Miner2, although the mammalian protein is distinct from Cox13 [86]. In *T. gondii*, ApiCox13 co-migrates with complex IV, promotes holoenzyme stability, and binds two Fe–S clusters, potentially suggesting a dual role in both structural maintenance and Fe–S cluster transfer [91].

## 5. *Plasmodium* iron proteome

We curated a provisional catalog of putative iron metalloproteins in *Plasmodium*, classified by cofactor type and supported by either direct evidence or conserved domain homology (S1 Table). Although this approach may overlook apicomplexan-specific proteins lacking clear homologs and cannot fully account for differences in metal cofactor usage across organisms, it establishes a valuable framework for defining the parasite iron proteome, pinpointing essential iron-dependent pathways, and providing context for modeling how parasites traffic and regulate cellular iron to ensure survival.

### 5.1. Mononuclear/Di-iron enzymes

We identified homologs of enzymes predicted to utilize at least one mononuclear iron atom, which are localized to diverse organelles to support a wide range of cellular functions. Among these proteins, a predicted prolyl hydroxylase–like protein (PF3D7_1445000) with only distant homology to other enzymes in this class is a noteworthy putative iron metalloprotein and is predicted to be essential for blood-stage growth. The *T. gondii* homolog (TGME49_214620) functions as an oxygen sensor that regulates protein translation under varying oxygen conditions, raising the possibility of a similar role in *Plasmodium* [93].

*Plasmodium* encodes only a handful of putative non-heme di-iron enzymes, but they support core processes such as translation, protein processing, and deoxyribonucleotide synthesis. These few enzymes are distributed across cellular compartments, including the cytosol, nucleus, endoplasmic reticulum, mitochondrion, and apicoplast, and most of these proteins are predicted to be essential during the blood stage [47,48,92].

### 5.2. Heme

*Plasmodium* encodes a small set of heme metalloproteins, most of which operate in the mitochondrial ETC. A notable outlier is cytochrome *c*-2 (PF3D7_1311700), a divergent homolog with an unknown function. It is dispensable in blood stages, yet its conservation across apicomplexans hints at an unrecognized aspect of mitochondrial biology in these organisms [30]. Outside the mitochondrion, the parasite expresses three *b*_*5*_-type cytochromes (PF3D7_1232300, PF3D7_1428700, PF3D7_0918100) that likely donate electrons to diverse metabolic pathways and/or help manage oxidative stress [94].

Multiple parasite-specific heme-binding proteins have been rigorously studied, including MSP3 (PF3D7_1035400), HRP2/3 (PF3D7_0831800, PF3D7_1372200), and HDP (PF3D7_1446800). These proteins have diverse proposed roles that include supporting parasite survival in a heme-rich environment by buffering excess heme, facilitating heme trafficking, supporting mitochondrial translation, and/or promoting heme crystallization into hemozoin [95–98].

### 5.3. Fe–S

Although *Plasmodium* retains only a small subset of heme proteins compared to other systems, the parasite encodes homologs of many Fe–S cluster proteins described in other eukaryotes. These essential Fe–S proteins are distributed across nearly all major metabolic organelles and rely on three distinct biogenesis pathways (Fig 3): the iron–sulfur cluster (ISC) machinery in the mitochondrion, the sulfur utilization factor (SUF) pathway in the apicoplast, and the cytosolic iron–sulfur assembly (CIA) system, which depends on the ISC pathway for a sulfur intermediate [82]. Several groups have highlighted both the unusual composition of these pathways in apicomplexan parasites and the striking absence of certain homologs, raising open questions about how Fe–S biogenesis is achieved in *Plasmodium* [71,82,99,100]. Overall, the central role of Fe–S biogenesis and metabolism in blood-stage parasites highlights the need for further studies to elucidate the organization and regulation of these pathways and to investigate the numerous putative Fe–S proteins that remain uncharacterized.

## 6. Conclusion

Over the past decade, remarkable progress has been made in defining the key players of iron metabolism during blood-stage *P. falciparum* infection. We now have a clearer picture of how the parasite acquires host iron, how specialized transporters sustain its intracellular economy, and how iron supports essential metabolic pathways. As highlighted in this review, several putative transporter and chaperone homologs remain uncharacterized and represent important priorities for future investigation.

At the same time, recent discoveries have further underscored that parasite iron homeostasis is strikingly distinct from model organisms. The absence of canonical ferric reductases and iron-responsive regulators suggests that *Plasmodium* relies on unconventional strategies to maintain cellular iron balance. The validated homeostatic proteins now provide a foundation for mapping these mechanisms, with approaches such as interaction proteomics and proximity biotinylation poised to uncover the wider network of parasite-specific modulators that govern iron levels.

Among the most pressing unanswered questions is how iron is trafficked into organelles with high metabolic demand, including the apicoplast and mitochondrion. No dedicated importers have yet been validated for these compartments. In addition, although the parasite can scavenge heme from host hemoglobin, the pathway by which heme is imported into the mitochondrion to support cytochrome function remains entirely unknown [30,31].

As elusive as iron regulation has been in malaria parasites, the field is now at an inflection point. With a growing set of validated iron homeostatic proteins serving as anchors and increasing cellular tools (e.g., genetically encoded iron sensors and proximity biotinylation probes), researchers are well-positioned to unravel how *Plasmodium* senses, traffics, and regulates iron across its subcellular compartments. Defining these pathways will deeply advance our understanding of parasite cell biology and unveil unique vulnerabilities in iron metabolism that may ultimately be leveraged against one of the deadliest pathogens in human history.

## Supporting information

S1 Table Proposed and characterized iron-containing proteins in *Plasmodium falciparum.* Proteins are classified as dispensable, fitness-conferring, or essential based on insertional mutagenesis studies [47,48,92].
(XLSX)
